# Reducing Length of Hospital Stay Following Transcatheter Aortic Valve Implantation

**DOI:** 10.3390/jcm13185433

**Published:** 2024-09-13

**Authors:** Ahmed R. Gonnah, Arif A. Khokhar, Ji-Jian Chow, Adam Hartley, Rahul Sethi, Saud Khawaja, Nearchos Hadjiloizou, Neil Ruparelia, Ghada Mikhail, Iqbal Malik

**Affiliations:** Hammersmith Hospital, Imperial College Healthcare NHS Trust, London W12 OHS, UK; ahmed.gonnah2@nhs.net (A.R.G.); g.mikhail@imperial.ac.uk (G.M.);

**Keywords:** TAVI, early discharge

## Abstract

Transcatheter aortic valve implantation (TAVI) has emerged as a safe and effective treatment for severe aortic stenosis across the spectrum of surgical risk cohorts. Subsequently, the dramatic increase in procedural volume worldwide has placed significant financial and logistical pressures on healthcare institutions, particularly regarding hospital length of stay (LOS), which can adversely affect patient flow. In this review article, we discuss different peri-procedural strategies developed to reduce LOS and facilitate early discharge after TAVI.

## 1. Introduction

Since the first TAVI procedure in 2002, the continuous evolution in device technology and procedural techniques has helped to transform TAVI into a safe, predictable, and effective solution for treating severe aortic stenosis [1,2,3,4]. Globally, TAVI procedural volume has dramatically increased because of the expanding indications, driven by an increasing body of evidence demonstrating the safety and efficacy of TAVI in high-, intermediate-, and low-surgical-risk cohorts [5,6,7,8]. This increase has placed significant financial and logistical pressures on healthcare systems, prompting the emergence of a more ‘minimalistic’ approach to TAVI. These refinements are designed to streamline the procedural workflow and reduce the peri-procedural complications, which together translate into a reduced length of stay (LOS) and earlier discharge [9]. Advances in procedural techniques, the use of electrophysiological testing, and telemonitoring are making early discharge after TAVI safer and more feasible [10,11,12,13]. Similar initiatives aimed at reducing the LOS after cardiac interventions have proven to be both feasible and safe [14,15]. Shortening the hospital LOS can alleviate the burden on healthcare systems, especially given the rising demand for TAVI procedures, without affecting the safety of patients. Cost savings come from reducing the use of hospital resources, such as bed occupancy, and potentially lowering the incidence of hospital-acquired complications [11,16].

In this review, we aim to synthesise existing literature to evaluate the key factors that facilitate early discharge after TAVI and to identify areas where further research is needed. By addressing this gap, we hope to provide a comprehensive overview of current best practices and highlight opportunities for future studies to further optimise post-TAVI care.

### 1.1. Procedural Refinements

The steady increase in TAVI procedural volume has resulted in many centres moving from performing TAVI under general anaesthesia (GA) to local anaesthesia (LA) with conscious sedation. In the SOLVE-TAVI (Comparison of Second-Generation Self-Expandable Versus Balloon-Expandable Valves and General Versus Local Anaesthesia in TAVI) randomised trial of 447 patients, the primary composite endpoint of all-cause mortality, stroke, myocardial infarction, infection requiring antibiotic treatment, and acute kidney injury, as well as the individual components, were similar between patients who underwent TAVI under LA and those receiving GA [17]. Additionally, multiple large-scale, multi-centre registries have consistently demonstrated the safety and efficacy of performing TAVI under LA, with the added benefit of a significantly reduced procedural duration and a reduction in hospital LOS [18,19,20]. A key concern when switching from GA to LA is the reduced utilisation of transoesophageal echo (TOE) and the impact this may have on paravalvular leak (PVL). However, improvements in valve design and technology, with the addition of sealing skirts in newer-generation devices, have ensured that rates of >mild PVL in contemporary TAVI remain reassuringly low [5,21].

The increased adoption of LA has in part been facilitated by improvements in the management of vascular access. Detailed pre-procedural computed tomography (CT) planning, a reduction in device profiles, the increased effectiveness of vascular closure devices (VCDs), and refinements in procedural techniques have helped to reduce the burden of major and minor vascular complications. These are often key contributors to hospital LOS. The routine use of ultrasound-guided vascular access during TAVI is associated with reduced major and minor vascular access site complications, earlier mobilisation, and a reduced LOS [22,23,24]. The use of dedicated VCDs for large-bore arterial access allows for safe and effective closure and can promote earlier mobilisation and recovery for patients [25,26]. The further use of ultrasound to guide access closure with the use of VCDs can further help to reduce the risk of vascular complications associated with VCD failure [27]. Switching from transfemoral to transradial secondary access has also been shown to reduce the rate of vascular complications [28,29]. In a propensity-matched analysis of 4949 patients, the use of transradial compared to transfemoral secondary access was associated with significant reductions in all vascular complications, major/life-threatening bleeding events, 30-day stroke rates, acute kidney injury, and overall mortality [30]. In addition to the favourable safety profile, transradial secondary access provides additional benefits in terms of patient recovery, earlier mobilisation, and potentially earlier discharge following the procedure.

Aside from vascular complications, the risk of conduction disturbances requiring permanent pacemaker implantation (PPI) can often account for significant delays in discharge. Often, patients who develop peri-procedural changes in conduction parameters require prolonged in-patient monitoring, which can delay discharge. Current guidance varies on what degree of conduction disturbance is deemed safe, further complicating decisions on safety for discharge [13,31]. Intra-procedurally, implantation depth and valve design are two factors which are strongly associated with the development of conduction disturbances. Procedural techniques aiming to systematically achieve higher implantation depths have been shown to reduce the risk of conduction disturbances with both balloon and self-expandable valves [32,33,34]. Using a right–left cusp overlap projection eliminates foreshortening of the left ventricular outflow tract, allowing for a more precise estimation of the implantation depth and permitting targeted higher implantations, particularly in patients at higher risk for developing conduction disturbances.

During TAVI, temporary pacing is usually required at the time of valve deployment or as a back-up in case of significant intra-procedural conduction disturbances or high-grade AV block. Conventionally, this necessitates the use of a temporary pacing wire inserted into the right ventricle (RV), which, due to its thinner walls, is at increased risk of perforation and subsequent pericardial tamponade. An alternative strategy for temporary procedural pacing is to use the left ventricular (LV) guidewire for pacing. LV, compared to RV, pacing is equally safe and effective during the procedure but is associated with a reduction in major vascular adverse events and pericardial tamponade [35,36,37].

Taken together, these procedural refinements, often described as ‘minimalist TAVI’, are designed to reduce vascular injury and conduction disturbances, resulting in significant improvements in procedural workflow, shorter LOS, and earlier discharge.

### 1.2. Early Discharge Pathways after TF TAVI

The significant increase in procedural volume has prompted the development of strategies and novel discharge pathways aimed at reducing the length of hospital stay (Table 1) [38,39,40,41,42,43,44,45,46,47,48,49]. The European TAVI Pathway survey of 147 centres across 26 countries performing over 27,000 TAVI procedures provides a snapshot of current contemporary practices. About 60% are performed under local anaesthesia with conscious sedation, 33% with local anaesthesia only, and 99% via percutaneous transfemoral access. Post-procedure, patients are often transferred to medium-care units (52%), high-care units (28%), or low-care units (20%), with discharge on day 1 for 12%, day 2 for 31%, day 3 for 29%, and day 4 or later for 28% [50]. This highlights how a significant proportion of patients are currently being considered suitable for early discharge after TAVI.

## 2. Feasibility, Safety, and Outcomes of Early Discharge after TAVI

Earlier studies first demonstrated the feasibility and safety of early discharge after TAVI. Barbanti et al. found that among 465 patients, 23.0% were discharged within 3 days, showing lower in-hospital bleeding, major vascular complications, and pacemaker implantation rates compared to late-discharge patients. There were no significant differences in 30-day outcomes such as death, bleeding, pacemaker implantation, or rehospitalisation [38]. Similar findings have since been replicated, confirming the relative safety of earlier versus later discharge after TAVI [40,42,45,48].

More recently, larger-scale studies such as the FAST-TAVI trial, Vancouver 3M Clinical Pathway, and FAST-TAVI II trial have demonstrated the effectiveness of implementing dedicated early discharge pathways in the contemporary era [43,44,49]. The FAST TAVI trial was conducted across multiple European countries, involving 502 patients from 10 sites. The study prospectively validated the adequacy of a set of discharge criteria in patients undergoing TF TAVI. The primary endpoint, a composite of adverse events at 30 days including mortality, vascular complications, permanent pacemaker implantation, stroke, rehospitalisation due to cardiac reasons, kidney failure, and major bleeding, occurred in 12.9% of patients. The overall 30-day mortality rate was low at 1.1%, with similar statistically low rates for all components. Patients appropriately discharged early had significantly lower risks of adverse events compared to those with delayed discharge, demonstrating the effectiveness of the pre-specified discharge criteria [43]. Additionally, the Vancouver 3M Clinical Pathway, which includes minimalist peri-procedural techniques, facilitated post-procedure recovery, and criteria-driven discharge achieved same/next-day discharge within 24 h (80.1%) and 48 h (89.5%), with excellent safety and efficacy outcomes [44]. This pathway was externally validated in Ontario, Canada, showing a low incidence of rehospitalisation (6.7%) and significant reductions in mechanical ventilation, surgical vascular cut-down, and hospital LOS. As a result, there was a significant increase in the number of patients receiving TAVI on a given procedural day from two to three patients in a relatively low-volume centre [46]. Moreover, seven centres used this validated Vancouver 3M Clinical Pathway to assess the efficacy and safety of same-day discharge in selected patients at low risk for adverse clinical events following discharge, showing feasibility, with the composite primary outcome of cardiovascular death, stroke, myocardial infarction, all-cause readmission, major vascular complications, and new permanent pacemaker (PPM) implantation at 30 days occurring in 5.7% patients [47]. There were also no major vascular complications, strokes, or deaths during the index admission. One patient (0.8%) required pacemaker implantation for complete heart block and was discharged the same day. Furthermore, no patient required a pacemaker between discharge home, and the 30-day follow-up [47]. Most recently, the FAST-TAVI II trial, evaluating 969 patients (mean age was 81.9 ± 6.6 years and mean EuroSCORE II was 4.4 ± 4.5%) from 10 TAVI centres in France, implemented quality care measures that led to a significantly higher early discharge rate (58.1%) compared to that in the control group (42.3%), with a reduction in median LOS and similar 30-day mortality and readmission rates [49]. These studies collectively demonstrate that with the right protocols, early discharge following TAVI is safe and feasible.

## 3. Predictors of Early and Late Discharge

The predictors of early and late discharge after TAVI include various demographic, clinical, and procedural factors. Demographic factors, including male sex and younger age, were also found to be associated with early discharge [41,51]. The presence of a pacemaker and the absence of general anaesthesia were associated with earlier discharge [38,39,42,51]. Additionally, the absence of acute kidney injury, atrial fibrillation, previous balloon aortic valvuloplasty, post-TAVI bleeding, and blood transfusion were independent predictors of early discharge [38,39,41,42,51]. Patients with a lower EUROSCORE, STS score, and baseline NYHA class < IV were discharged earlier [38,42,48]. In addition, the use of self-expandable valves was associated with later discharge [51]. Collectively, these findings emphasise the importance of patient selection and adherence to validated discharge criteria to ensure safe and effective early discharge following TAVI [38,39,40,41,42,43,44,45,46,47,48,49,50,51,52,53]. In many instances, the LOS in hospital is prolonged for no apparent reason compared to that in a similar-volume centre, and hence, efforts should be made to educate centres to reduce LOS [54]. These findings underscore the importance of quality improvement initiatives in optimising outcomes and resource utilisation within the context of TAVI. Ultimately, these insights contribute to the ongoing evolution of post-TAVI care. Table 2 summarises the factors associated with late discharge based on the current evidence.

### 3.1. Electrophysiological Studies to Predict Conduction Abnormalities and Need for Pacemaker Implantation

Conduction disturbance after TAVI is one of the major contributors to delayed hospital discharge. Pre-existing right bundle branch block is strong predictor of pacing requirement after TAVI; first degree heart block has been implicated but is a less reliable predictor [12]. The observations that (A) many patients with pre-existing RBBB do not develop high-grade atrioventricular block (HG-AVB) and (B) those that have prophylactic pacemakers in this scenario rarely receive a high pacing burden have led to the European Society of Cardiology recommending against the blanket implantation of devices before TAVI [31]. The American College of Cardiology recommends that available pre-TAVI Holter data be reviewed for conduction disease warranting a permanent device irrespective of TAVI, but there is no consensus on whether continuous ECG should always be performed prior to TAVI and, if so, for how long [12].

Following TAVI, standard pacemaker indications such as complete heart block and Mobitz II AV block warrant permanent implants along the same guidelines as for non-TAVI patients. Alternating left and right bundle branch block also warrants pacemaker implant after TAVI. New post-TAVI conduction system disease such as PR prolongation or new bundle branch block attracts ambulatory monitoring recommendations from both European and American guidelines, which is generally at least 48 h—a significant addition to discharge time [12,31].

Given the limitations of body surface ECG in the early prediction of conduction disturbance for TAVI patients, there has been interest in invasive electrophysiology and its potential role in identifying those in need of permanent pacemakers. In non-TAVI patients with bundle branch block, the His-Q interval is already known to be a significant predictor of high-degree AV block, displaying an increased risk for those with an His-Q value ≥ 70 ms, with His-Q ≥ 100 ms being identified as particularly high risk [55]. There have been several studies implementing different invasive electrophysiological tests to predict conduction abnormalities such as HG-AVB and bundle branch block, which commonly occur after TAVI; these can help determine the need for PPM implantation [56,57,58,59,60].

Kostopoulou et al. described the use of a pre-TAVI electrophysiological study which was repeated 2 days after TAVI to determine predictors for PPM implantation in 45 CoreValve recipients [56]. The atrial-His interval (AH) and His-ventricular (HV) intervals, effective refractory period (ERP) of the atrioventricular (AV) node, and Wenckebach cycle length (WCL) were measured [31]. Pacemakers were implanted for complete AV block, Mobitz II, and new LBBB with a HV interval >70 ms; 24% of patients received pacemakers. Patients with a prolonged HV interval prior to procedure had a significantly higher risk of pacemaker implantation, but the proposed cut-off of 52 ms from receiver operating characteristic (ROC) analysis was non-significant (*p* = 0.08). Long-term follow-up revealed few remained pacemaker dependent. In the two patients with long-term dependency, changes in the HV interval were minimal before and after the procedure (0 and +9 ms) [56].

Knecht et al. prospectively analysed patients with pre-existing (25% of the cohort) or new LBBB following TAVI, invasively measuring the HV interval within 24 h of valve implant [57]. Patients with a normal HV interval (≤ 55 ms) received a loop recorder (ILR-group), while those with a prolonged HV interval (>55 ms) underwent pacemaker implantation (PM-group). The primary endpoint was the occurrence of HG-AVB during a 12-month follow-up. Among 56 patients included, 10% in the ILR-group vs. 53% in the PM-group (*p* < 0.001) experienced HG-AVB (or ventricular pacing > 1% in the PM group). Despite non-significant differences in surface markers, the HV interval did predict HG-AVB. An HV interval >55 ms yielded a negative predictive value of 90%, suggesting that new LBBB patients with a short HVI could be candidates for early discharge.

The LBBB-TAVI study prospectively examined patients with new LBBB following TAVI with an electrophysiology study followed by the implantation of a dual-chamber pacemaker in high-risk patients (His-ventricle interval ≥ 70 ms) or an implantable loop recorder in the lower-risk group, with a 12-month follow-up. An HV interval ≥ 70 ms following TAVI had a specificity of 83% and sensitivity of 44% for HG-AVB at 12 months, further supporting the safe discharge of short-HVI patients. Notably, a QRS duration > 150 ms also predicted HG-AVB in this study, but a sensitivity analysis was not performed on body surface markers [58].

Krishnaswamy et al. assessed the use of rapid atrial pacing and the detection of Wenckebach at up to 120 bpm in 284 patients [59]. About 45% of patients had Wenckebach, and the negative predictive value for pacemaker implantation was 97%. Pacemakers were mainly implanted for complete heart block (65%), with broadening LBBB/PR interval as the next most common reason (23%). Notably, 6% of pacemakers were implanted for sinus node dysfunction, which is neither affected by the AV node nor assessable by Wenckebach point. Also, LBBB on surface ECG prior to TAVI was a significant predictor of pacemaker implant, overlapping the confidence intervals of the EP study, but the authors did not provide a head-to-head comparison.

Ferreira et al. performed pre- and 4–5-day post-TAVI EP study in 74 TAVI recipients, 28% of whom received permanent pacemakers for Mobitz II and complete heart block prior to discharge [60]. In those with immediate complete heart block, temporary pacing was used until day 5, but the number of patients recovering conduction in that time was not stated. Surface PR, QRS duration, and AH and HV interval were found to be significant univariate predictors of pacemaker implant, but only HV interval was significant in multivariate analysis. An attempt to define a high-risk cut-off showed that HV = 65 ms was significant (*p* = 0.02) following TAVI and insignificant prior to TAVI (*p* = 0.11) in univariate analysis, but ROC analysis was not performed. This study is notable because in the other studies comparing pre- and post-TAVI EP studies, the pre-TAVI study was helpful, where in this study, it was not.

Finally, a meta-analysis of 18 studies involving 1230 patients evaluated the value of electrophysiological studies in predicting HG-AVB and guiding PPM implantation after TAVI [61]. Overall, the rate of PPM implantation for HG-AVB was 16%. After TAVI, atrioventricular conduction intervals, particularly AH and HV intervals, were longer than those prior to TAVI, but the numbers of patients crossing a threshold of abnormality were not quantified. A pre-TAVI HV >70 ms and absolute post-TAVI HV interval (continuous variable, no cut-off defined) were associated with subsequent HG-AVB and PPM implantation. The study outlined that selective electrophysiological testing could aid in the risk stratification of post-TAVI HG-AVB and guide PPM implantation, particularly in patients with equivocal indications, with a 57% long-term PPM dependency rate.

Although these studies suggest the importance of EP testing in guiding the need for pacemaker implantation following TAVI, performing an EP study is not without consequences. The need for an additional invasive procedure, the impact on delaying discharge, and overall additional healthcare costs all need to be considered. A cost–benefit analysis for the lifetime management of aortic stenosis could be considered whereby the upfront costs of an additional procedure are balanced against the costs and consequences of implanting a pacemaker, especially when the pacing burden is likely to be minimal [62,63]. Additionally, it remains to be seen whether invasive EP measurements can surpass the predictive accuracy and sensitivity of body surface ECG in the early identification of patients who require pacemaker implantation.

### 3.2. Telehealth/Monitoring after TAVI

The implementation of an organised fast-track consultation system, offering each patient early personalised access to healthcare support through various methods after discharge, could have a significant positive impact. This approach would likely enhance post-discharge care by providing timely medical advice, addressing patient concerns, and potentially preventing unnecessary readmissions. Additionally, such accessibility could improve patient confidence and satisfaction, ensuring that patients feel supported during their recovery. By offering a structured, individualised follow-up mechanism, whether via telemedicine, phone consultations, or in-person visits, healthcare providers can better manage post-discharge complications while further alleviating pressure on hospital services. They can effectively offer the option for longer-term monitoring to assess for conduction abnormalities remotely and hence determine the patient groups that may benefit from pacing. The recently conducted TELE-ACS trial (a randomised clinical trial exploring the effect of a telemedicine-based approach on managing patients following acute coronary syndrome), demonstrated the feasibility of this approach, coupled with a significant reduction in hospital readmission and patient-reported symptom burden [64]. Thus, the prospect of remote patient monitoring to facilitate early discharge from hospital following TAVI is an alluring one. The most obvious strategy to support early discharge after TAVI procedures involves remote electrocardiographic (ECG) telemetry in an attempt to limit the in-hospital monitoring that is usually mandated following valve deployment, especially with self-expanding devices. To this end, several non-randomised studies have utilised ambulatory ECG monitoring, for which many devices are available, to detect indications for pacing. Examples of devices include ECG patches such as the Zio patch [65] (rhythm monitoring for 14 days, but data can only be reviewed retrospectively), the SHL Telemedicine SmartHeart [66] (12-lead ECG when activated and worn by the patient), the Smartcardia [67] (7-lead ECG with up to 14 days of remote monitoring), and the Fourth Frontier [68] (unlimited single-lead ECG but with device removal for charging). An example of one such clinical study is the REdireCT TAVI prospective cohort study that utilised 2 weeks of ambulatory ECG monitoring to detect patients requiring permanent pacemaker implantation [69].

Another interesting approach has been to utilise ambulatory ECG monitoring prior to TAVI to further risk stratify patient pacing requirements [70]. Remote ECG monitoring coupled with other vital sign monitoring, potentially with artificial intelligence-facilitated event detection, has the potential to provide feasible, sustainable, and cost-effective remote healthcare. When this is combined with patient-activated alerts and readily available physician access (for example via telephone or video consultation), there is the opportunity to safely provide hospital-grade monitoring outside of a clinical setting, thus supporting and facilitating early discharge after TAVI [71].

### 3.3. Future Perspectives

As the TAVI procedural volume is expected to continually increase, further efforts to reduce the impact on healthcare institutions is warranted. Improvements in device technology aimed at reducing vascular complications with smaller sheath profiles and minimising conduction disturbances with valve design are expected. Furthermore, existing and future early discharge pathways merit careful evaluation to determine their safety and effectiveness given the heterogeneity of the studied populations. The aforementioned studies predominantly included low-risk patients with favourable health profiles, limiting the generalisability of the findings to higher-risk populations, such as elderly individuals or those with multiple comorbidities. Most studies had short follow-up periods, typically limited to 30 days, which may not capture the long-term effects of early discharge, such as late complications or survival rates. Additionally, a potential selection bias is present, as patients with complications or pre-existing conditions were often excluded, possibly overestimating the safety of early discharge. The variability in discharge criteria and institutional protocols further limits the comparability of findings. Lastly, most studies were conducted in high-volume centres with specialised care, which may not reflect outcomes in lower-volume or resource-limited settings, reducing the external validity of the results. These limitations highlight the need for further research to assess the broader applicability and long-term outcomes of early discharge following TAVI. This will also allow for the appropriate selection of patients for safe early discharge.

## 4. Conclusions

**Figure 1:**
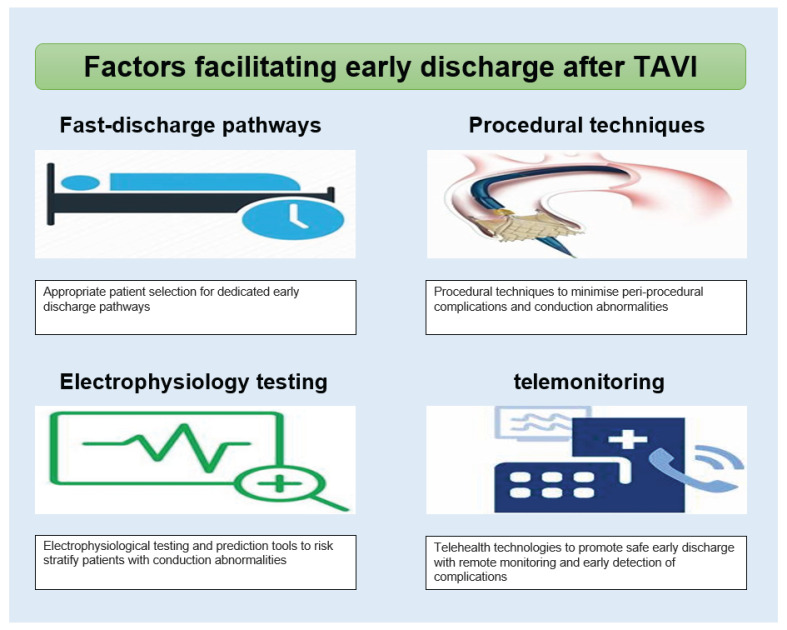
Graphical illustration of the factors facilitating early discharge after TAVI.

In conclusion, safe early discharge after TAVI can be achieved by refining procedural techniques and adopting validated discharge protocols. Electrophysiological assessments help to identify patients at risk of conduction disturbances who may require extended monitoring and can identify the appropriate patient cohort that can benefit from permanent pacemaker implantation. Limitations remain, as this can increase costs and delay discharge, which warrants the need for further dedicated trials to compare its efficacy to surface ECG monitoring. Integrating telehealth into post-discharge care enables continuous monitoring and early intervention, ensuring patient safety while supporting earlier discharge. These combined strategies offer a comprehensive approach to optimising patient outcomes following TAVI. A summary of our findings is displayed in Figure 1.

## Figures and Tables

**Table 1:** Summary of the studies that implemented an early discharge pathway after undergoing TAVI [38,39,40,41,42,43,44,45,46,47,48,49].

Author	Country	Year	Discharge Pathway	Number of Patients (*n*)	Methods	TAVI Prosthesis	Criteria for Fast-Track Discharge	Outcomes
Barbanti et al. [38]	Italy	2015	Early discharge (**within 72 h**) vs. late discharge (**after 3 days**) following TF TAVI	Unmatched population (*n* = 465) Matched population (*n* = 267)	Unmatched population: early discharge (*n* = 107, 23.0%), late discharge (*n* = 358, 77.0%) Matched population: early discharge (*n* = 89, 33.3%), late discharge (*n* = 178, 66.7%)	Unmatched population: Edwards–SAPIEN (*n* = 127), CoreValve (*n* = 331), Lotus (*n* = 3), Portico (*n* = 2) Matched population: Edwards–SAPIEN (*n* = 78), CoreValve (*n* = 186), Lotus (*n* = 1), Portico (*n* = 2)	Suitability for discharge was determined by the attending physician and by the TAVI operator in accordance with the clinical status of the patient and the procedural outcomes (e.g., absence of conduction disturbances, bleeding, and the need for a pacemaker).	Unmatched population: New York Heart Association (NYHA) Class IV (OR: 0.22, 95% CI 0.05 to 0.96; *p* = 0.045) *, any bleeding (OR: 0.31, 95% CI 0.74 to 0.92; *p* = 0.031) * Matched population: in-hospital bleeding (7.9% vs. 19.4%, *p* = 0.014) *, major vascular complications (2.3% vs. 9.1%, *p* = 0.038) *, pacemaker implantation (7.9% vs. 18.5%, *p* = 0.021) * At 30 days, death (2.2% vs. 1.7%, *p* = 0.540), bleeding (0.0% vs. 1.1%, *p* = 0.444), pacemaker implantation (1.1% vs. 0.0%, *p* = 0.333), and rehospitalisation (1.1% vs. 1.1%, *p* = 1.000)
Durnand et al. [39]	France	2015	Early discharge (**within 72 h**) vs. late discharge (**after 3 days**) following TF TAVI	337	Early discharge (*n* = 121, 35.9%), Late discharge (*n* = 216, 64.1%)	Edwards SAPIEN-XT (*n* = 337)	Patients were eligible for early discharge if the procedure was not performed in an emergency and if there were no major complications during the procedure and the first 24 h of monitoring in ICU (i.e., stroke, myocardial infarction, major vascular complication requiring transfusions, life-threatening bleeding, or acute kidney injury stage 2 or 3). Careful electrocardiographic monitoring in the ICU allowed for early conduction disturbance detection. A pacemaker was systematically implanted in patients with persistent complete atrioventricular block 24 h after TAVI. Patients with persistent de novo complete left bundle branch block were also closely monitored and were not eligible for early discharge.	Primary end point: combined death and rehospitalisation at 30-day follow-up (3.3% vs. 5.1%, *p* = 0.58)
Noad et al. [40]	United Kingdom	2016	**Same/next day** vs. **early discharge** (**within 72 h**) vs. **late discharge** (**after 3 days**) following TF TAVI	80	Same/next day (*n* = 25, 21.7%), early discharge (*n* = 39, 32.5%), late discharge (55, 45.8%)	Medtronic (*n* = 51), Edwards (*n* = 34), St Jude Medical (*n* = 18), Boston Scientific (*n* = 17)	Post-operative criteria for early discharge included no evidence of respiratory distress, apyrexia, stable bloods, no conduction abnormalities, wires/sheaths removed, wounds satisfactory—no evidence of bleeding, mobilising, and a satisfactory post-op echocardiogram.	30-day mortality (0% vs. 0% vs. 5.45%, *p* = 0.167), readmission rates between groups (3.84% vs. 5.12% vs. 5.45% *p* = 0.952) Resource analysis revealed the late discharge group cost £3091.6 more per patient per TAVI than same/next-day discharge group.
Kamioka et al. [41]	United States	2018	**Next-day** vs. **non-next-day** discharge after TF-TAVI	360	Next-day (*n* = 150) vs. non-next-day (*n* = 210) discharge	SAPIEN-XT (*n* = 99), SAPIEN 3 (*n* = 261)	Patients with no complications as per Valve Academic Research Consortium-2 (VARC-2) criteria, including vascular complications, bleeding, stroke, and a new complete heart block, or post-procedural complications were eligible for next-day discharge.	The 30-day composite outcome (hazard ratio: 0.62; 95% CI: 0.20 to 1.91) was similar in both groups. The composite outcome at 1 year was significantly lower in the next-day discharge group (hazard ratio: 0.47; 95% CI: 0.27 to 0.81) *.
Aldalati et al. [42]	United Kingdom	2018	Early discharge (**within 72 h**) vs. late discharge (**after 3 days**) following TF TAVI	319	Early discharge (*n* = 56, 17.5%), late discharge (*n* = 263, 82.5%)	SAPIEN-XT (*n* = 248), SAPIEN 3 (*n* = 60)	Patients were routinely monitored in the intensive care unit after TAVI. A minimum 24 h stay in a level-two ward followed by a step down to a cardiology/cardiothoracic ward (level one) with early mobilisation and physiotherapy. On day one or two (day of index procedure is day 0), patients underwent transthoracic echocardiography. If clinically stable, the majority of patients were considered for planned discharge on day two.	There was no statistical difference in terms of Valve Academic Research Consortium-2 (VARC-2) criteria outcomes, all-cause readmission rates, and the need for permanent pacemaker implantation. Mortality at 1 year was better among the early discharge group (3.6% vs. 15.6%, *p* = 0.014) *.
Barbanti et al. [43]	Italy	2019	**≤24 h, ≤48 h, ≤72 h, >72 h** discharge after TF-TAVI	496	≤24 h (133, 26.8%), ≤48 h (253, 51.0%), ≤72 h (360, 72.6), >72 h (136, 27.4%)	SAPIEN 3 (*n* = 496)	Early discharge criteria included NYHA Class ≤II, no chest pain attributable to cardiac ischaemia, no untreated major arrhythmias, patients having complications on day 0 to 1 but free of signs or symptoms on day 3, no fever during the last 24 h and no signs of an infectious cause, independent mobilisation and self-care, preserved diuresis (>40 mL/h during the preceding 24 h), no unresolved acute kidney injury type 3 (according to VARC-2 criteria), no red blood cell (RBC) transfusion during the preceding 72 h, stable haemoglobin in two consecutive samples (defined as a decrease of no more than 2 mg/dl), no paravalvular leak (PVL) with aortic regurgitation less than moderate, no stroke/transient ischaemic attack (TIA), and no haemodynamic instability.	The primary endpoint was defined as a composite of all-cause mortality, vascular access-related complications, pacemaker implantation, stroke, rehospitalisation due to cardiac reasons, kidney failure, and major bleeding at 30 days (7.0 vs. 26.4%; *p* < 0.001) *, which was reflected in some of its relevant components: stroke (0.0 vs. 2.8%; *p* = 0.015) *, permanent pacemaker implantation (4.3 vs. 15.9%; *p* < 0.001) *, major vascular complications (0.3 vs. 4.7%; *p* = 0.004) * and major/life-threatening bleeding (0.3 vs. 6.5%; *p* < 0.001) *.
Wood et al. [44]	Canada and the United States	2019	Discharge **within ≤24 h and ≤48 h** was tested by following the implementation of the Vancouver 3M Clinical Pathway for TF TAVI.	411	Of 1400 screened patients, 411 were enrolled at 13 centres and received a SAPIEN XT (58.2%) or SAPIEN 3 (41.8%) valve at low- (*n* = 148), medium- (*n* = 80), and high- (*n* = 183) volume centres.	SAPIEN-XT (*n* = 239), SAPIEN 3 (*n* = 172)	Criteria-driven discharge was based on three aspects: monitoring, facilitated recovery, and communication. Monitoring included a satisfactory echocardiogram and an absence of conduction delay, vascular access complications, and laboratory contraindications. Facilitated recovery was defined as a return to baseline mobilisation, absence of elimination issues, and return to baseline hydration. The multidisciplinary agreement of safety for discharge, a review of the discharge plan with family, and the availability of adequate follow-up appointments facilitated early discharge.	The primary outcomes of a composite of all-cause death or stroke by 30 days occurred in 2.9% (95% confidence interval: 1.7% to 5.1%), with neither component of the primary outcome affected by hospital TAVR volume (*p* = 0.51). Discharge was achieved in patients within ≤24 h (80.1%) and in patients within ≤48 h (89.5%).
Baekke et al. [45]	Denmark	2020	Fast-track (**3 days**, ^IQR 2–4) vs. standard discharge (6 days, ^IQR 4–8) in patients undergoing TF TAVI	914	Fast-track (*n* = 456) August 2015 to August 2017 vs. standard discharge (*n* = 458) August 2011 to August 2015	Before propensity score matching (PSM) (*n* = 864): balloon expandable (*n* = 99), mechanically expandable (*n* = 94), self-expandable (*n* = 721) After propensity score matching (PSM) (*n* = 628): balloon expandable (*n* = 65), mechanically expandable (*n* = 55), self-expandable (*n* = 518)	Fast-track discharge was determined by the evaluation of peri-procedural and short-term ECG dynamics as to determine patients with a low risk of needing a permanent pacemaker regardless of the presence of a pre-procedural bundle branch block.	All-cause mortality at 30-day (1.3% vs. 1.9%, *p* = 0.52) and 90-day follow-up (2.9% vs. 4.1%, *p* = 0.42) New permanent pacemaker implantation at 30-day (15.8% vs. 21.2%, *p* = 0.16) and 90-day follow-up (15.8% vs. 21.9%, *p* = 0.12) Rate of rehospitalisation between discharge and 90-day follow-up (2.09 per patient-year vs. 2.09 per patient-year, *p* = 0.99)
Hanna et al. [46]	Canada	2022	The Vancouver 3M Clinical Pathway for TF TAVI	291	291 patients underwent TAVI between 2012 and 2021.	Balloon expandable (*n* = 185), self-expanding system (*n* = 107)	Criteria-driven discharge was based on three aspects: monitoring, facilitated recovery, and communication. Monitoring included a satisfactory echocardiogram and an absence of conduction delay, vascular access complications, and laboratory contraindications. Facilitated recovery was defined as a return to baseline mobilisation, absence of elimination issues, and return to baseline hydration. The multidisciplinary agreement of safety for discharge, a review of the discharge plan with family, and the availability of adequate follow-up appointments facilitated early discharge.	Rehospitalisation within 30 days (*n* = 11, 6.7%) The need for mechanical ventilation and surgical vascular cut-down declined from 100% and 97%, respectively, at baseline, to 6% and 2% *. The number of patients receiving TAVI on a given procedural day increased from two to three patients * The hospital length of stay decreased from 5 days (2–6 days) to 1 day (1–3 days) *.
Barker et al. [47]	The United Kingdom, Canada, and the United States	2022	**Same-day** discharge vs. **non-same-day** discharge	2100	Same-day discharge (*n* = 124) vs. non-same-day discharge (*n* = 1976)	Balloon expandable (*n* = 120), self-expandable (*n* = 4)	The Multicenter PROTECT TAVR Study patient selection criteria for same-day discharge were at the discretion of the local multidisciplinary heart team across seven international sites. The study used selected elements of the previously validated Vancouver 3M clinical care pathway.	There were no major vascular complications, strokes, or deaths during the index admission. One patient (0.8%) required PPM implantation for complete heart block and was discharged the same day. No patient required a PPM between discharge home and the 30-day follow-up. The composite of cardiovascular death, stroke, myocardial infarction, all-cause readmission, major vascular complications, and new PPM at 30 days occurred in 5.7% patients (*n* = 6 of 106).
Angelillis et al. [48]	Italy	2022	Fast track (**≤3 days**) vs. slow track (**≥3 days**) following TF-TAVI	1501	Fast track (*n* = 224) vs. slow track (*n* = 1277)	EVOLUT R (*n* = 1068), EVOLUT PRO (*n* = 433)	The aim of this analysis was to compare the ‘Fast-Track’ population, with a post-procedural LOS less than or equal to 3 days, vs. the ‘Slow-Track’ population, with a post-procedural LOS greater than 3 days, in terms of demographic, baseline and procedural characteristics, procedural complications, and short-term and long-term clinical and echocardiographic outcomes, obtained at the follow-up clinic visit.	Death (1.8% vs. 0.6%, *p* = 0.09), cardiovascular rehospitalisation (1.3% vs. 1.6%, *p* = 0.99) at 30-day follow-up Death (4.0% vs. 6.9%, *p* = 0.10), cardiovascular rehospitalisation (3.6% vs. 8.1%, *p* = 0.01) * at 1-year follow-up
Durand et al. [49]	France	2024	Early discharge (**≤3 days**) and late discharge (**≥3 days**) were evaluated in patients undergoing the FAST-TAVI II pathway, which includes 10 quality-of-care measures for TF-TAVI.	1829	Intervention group (*n* = 969) vs. control group (*n* = 860)	SAPIEN (*n* = 923), CoreValve (*n* = 480), Acurate (*n* = 106), Portico (*n* = 47)	The intervention consisted of the implementation of 10 quality-of-care measures, including 5 logistical measures and 5 preventive measures to reduce LOS after TF TAVI. Logistical measures were the education of patients, education of medical teams, daily monitoring of patients, anticipation of post-TAVI transthoracic echocardiography, and early mobilisation. Preventive measures were a decision tree for the management of conduction disturbances, 12 echo-guided or angio-guided femoral puncture, echographic or angiographic control of femoral closure, prevention of bleeding, and prevention of acute kidney injury.	Early discharge was achieved in 563 (58.1%) patients in the intervention group vs. 364 (42.3%) patients in the control group (*p* < 0.0001) *. The median length of stay was significantly reduced in the intervention group compared to the control group [3 (IQR: 3) vs. 4 days (IQR: 3), *p* < 0.0001] *. Thirty-day mortality was low and similar in the two groups (0.5% vs. 0.9%, *p* = 0.30), as were 30-day readmissions (4.6% vs. 2.8%, *p* = 0.28).

All odds ratios and outcomes were relative to the early discharge pathway; * denotes outcome statistically significant favouring the early discharge pathway. ^IQR = interquartile range.

**Table 2:** Summary of the predictors of late discharge following TAVI [38,39,41,42,51].

Predictors of Late Discharge Following TAVI		
Demographic	Clinical	Procedural
Female sex	Absence of previous PPM	General anaesthesia
Older age	Post-procedural bleeding	Use of self-expandable valves
	Post-procedural blood transfusion	
	Acute kidney injury	
	Atrial fibrillation	
	Previous balloon aortic valvuloplasty	
	Higher EUROSCORE	
	Higher STS score	
	Baseline NYHA Class IV	
